# Altered Mucins (MUC) Trafficking in Benign and Malignant Conditions

**DOI:** 10.18632/oncotarget.2370

**Published:** 2014-08-26

**Authors:** Suhasini Joshi, Sushil Kumar, Amit Choudhury, Moorthy P. Ponnusamy, Surinder K. Batra

**Affiliations:** ^1^ Department of Biochemistry and Molecular Biology, University of Nebraska Medical Center, Omaha, NE, 68198, U.S.A; ^2^ Momenta Pharmaceuticals, Inc. Cambridge, MA 02142, USA; ^3^ Fred and Pamela Buffett Cancer Center, Eppley Institute for Research in Cancer and Allied Diseases, University of Nebraska Medical Center, Omaha, NE 68198, U.S.A.

**Keywords:** Mucins, cancer, trafficking, endocytosis, plasma membrane

## Abstract

Mucins are high molecular weight O-glycoproteins that are predominantly expressed at the apical surface of epithelial cells and have wide range of functions. The functional diversity is attributed to their structure that comprises of a peptide chain with unique domains and multiple carbohydrate moieties added during posttranslational modifications. Tumor cells aberrantly overexpress mucins, and thereby promote proliferation, differentiation, motility, invasion and metastasis. Along with their aberrant expression, accumulating evidence suggest the critical role of altered subcellular localization of mucins under pathological conditions due to altered endocytic processes. The mislocalization of mucins and their interactions result in change in the density and activity of important cell membrane proteins (like, receptor tyrosine kinases) to facilitate various signaling, which help cancer cells to proliferate, survive and progress to more aggressive phenotype. In this review article, we summarize studies on mucins trafficking and provide a perspective on its importance to pathological conditions and to answer critical questions including its use for therapeutic interventions.

## INTRODUCTION

Mucins (MUC) are high molecular weight O-glycoproteins, predominantly expressed at the apical surface of the epithelial cells [1-4]. Tissue specific expressions of MUC have essential functions to provide protection, lubrication to epithelial cells, maintenance of epithelial characteristics, cellular adhesion, differentiation, and immunity [1-5]. The expression of MUC is significantly altered during tumorigenesis and other pathological conditions. For example, MUC4 is not expressed in the normal pancreas, but the early pancreatic intraepithelial neoplasia (PanINs) precursor lesions have been shown to express MUC4, which further increases as the disease progresses [4-6]. In addition, MUC4 is also overexpressed in breast, gastric and ovarian cancer [7-9], and its overexpression has been associated with the poor prognosis of pancreatic cancer and cholangiocarcinoma [10, 11]. However, MUC4 expression is down-regulated during prostate carcinomas [12] and urothelial cancer [13], suggesting the complicated context-dependent role of mucins. Another example, MUC1 is overexpressed in various malignancies and inflammatory conditions [1, 14-16]. Besides the aberrant overexpression of MUC, emerging evidence suggests that anomalies in their subcellular localization and resultant changes in their endocytic trafficking play critical roles under pathological conditions [17].

**Figure 1:**
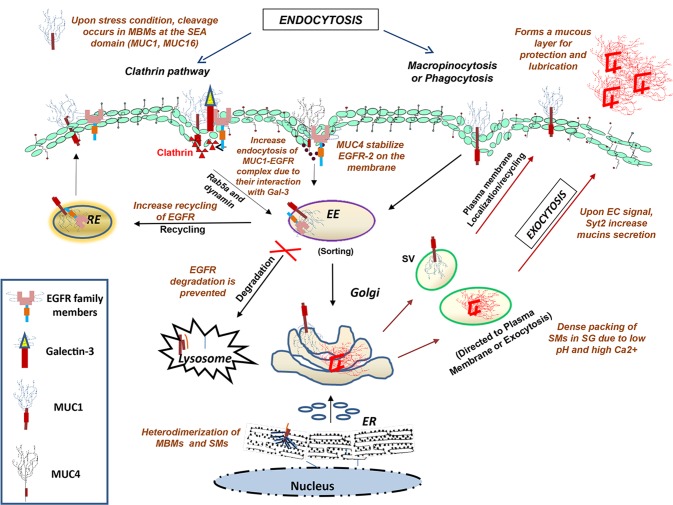
Diagrammatical representation of the intracellular transport of glycoproteins along endocytic and exocytic pathways Internalization of cell surface glycoproteins occurs by clathrin-mediated, caveolin-mediated, or clathrin- & caveolinindependent pathways, followed by the fusion of internalized vesicles with early endosomes where the cargo is sorted and targeted for either recycling (from trans-Golgi, late endosome and recycling endosome) or for degradation (in lysosomes). The other exocytic route are representative of the secretory pathways, where glycoproteins are first synthesized and processed in the rough ER followed by their entry into the Golgi, where they are further modified, packaged into secretory vesicles (SV) and either targeted to the plasma membrane or secreted by the exocytic machinery. **Abbreviations:** EE, early endosome; RE, recycling endosome; MBMs, membrane bound mucins; SMs, secretory mucins; SV, secretory vesicles; SG, secretory granules; EC, extracellular

In a cell, majority of proteins are not pre-set to any single location and are in a steady-state distribution due to opposing egress (exocytosis) and entrance (endocytosis) pathways [18]. These two pathways are extremely dynamic and are regulated by highly sensitive cross talks between different subcellular compartments. Endocytic pathways have always been considered as enduring mechanisms for recycling molecules from the plasma membrane to different intracellular compartments, and reduce receptor density at the cell surface resulting in signal attenuation. Proteins could be endocytosed by utilizing clathrin-mediated pathway, caveolae-mediated pathway, macropinocytosis, and phagocytosis [19]. The MUC1 utilizes these pathways for endocytosis and cell surface localization [20-22] (**Fig. 1**). Like other glycoproteins, MUC are also sorted after their internalization in the early or sorting endosome, where their fates are decided including their recycling, transportation to the Golgi (retrograde), and proteosomal or lysosomal degradation. This is not only responsible for efficient and regulated cellular metabolism and signal transduction, but is also required for coordinating the functions of each intracellular compartment by maintaining their specific compositions. Intriguingly, the trafficking of MUC and other glycoproteins is mainly regulated by post-translational modifications, including phosphorylation, glycosylation, palmitoylation and ubiquitylation.

In this review article, we provide a perspective on MUC trafficking and its pertinence to pathological conditions and discuss critical issues surrounding its potential use for therapeutic interventions.

## MUC BIOSYNTHESIS AND MECHANISM OF THEIR SECRETION

MUC are broadly classified into two categories: membrane bound mucins and secretory mucins. Membrane bound mucins (such as MUC1, MUC3, MUC4, MUC16, MUC17) have the ability to tether themselves into the plasma membrane due to the presence of transmembrane domain, whereas secretory mucins (such as MUC2, MUC5AC, MUC5B) get packaged into secretory vesicles and released upon receiving appropriate extracellular signal (**Fig. 1**) [4]. MUC have different physicochemical properties due to the presence of different domains like nidogen-like domain (NIDO), sea urchin sperm protein-enterokinase-agrin (SEA), von Willebrand factor D domain (vWD) and epidermal growth factor (EGF)-like domain. All the MUC are translated as a single polypeptides chain and are processed into rough endoplasmic reticulum and Golgi complex to acquire post-translational modifications, predominately N- and O-linked glycosylation.

Apart from glycosylation, some MUC are cleaved at specific proteolytic cleavage sites during post-translational processing, which create two subunits that remain associated throughout their transport from endoplasmic reticulum to Golgi complex and finally to the cell surface. MUC, including MUC1, MUC3, MUC16, and MUC17 are cleaved at their evolutionary conserved SEA domain by an unidentified intracellular protease, changes in pH, ionic concentrations and mechanical stress [4,23,24]. The cleaved extracellular domains (ECD) are shedded to rapidly clear cell surface associated materials or colonized/associated pathogens [25,26]. MUC that do not have the SEA domain e.g., MUC2, MUC4, MUC5AC are postulated to get cleaved at their GDPH (Gly-Asp-Pro-His) sequence [27-29]. The substitution of the aspartic residue with glutamic residue in the GDPH sequence, which leads to the lengthening of the side chain by one carbon, has shown to abolish the cleavage [29]. Acidic environment of endoplasmic reticulum plays an important role in GDPH cleavage, though different MUC require different pH for the cleavage. For example, MUC2 cleavage is stimulated at pH less than 6 [27], whereas MUC5AC cleavage has been demonstrated to occur at neutral pH of endoplasmic reticulum [29]. Nevertheless, the cleavage of the MUC5AC mucin is augmented at lower pH.

The pathways of MUC trafficking are largely similar for both secretory and membrane bound MUC, and the slight differences in their trafficking occur due to variability in their domains. For instance, dimerization of MUC2 occurs in the endoplasmic reticulum due to formation of disulfide bonds in the cysteine knot domain [30] and the subsequent trimerization occur by forming disulfide bonds in the vWD3 domain in TGN [31]. MUC2 and possibly other gel forming secretory MUC form large net-like structures, required to protect epithelia from various harsh conditions. The dense packing of MUC2 in secretory granules occurs due to the formation of large aggregates under high Ca^2+^ and low pH conditions (**Fig. 1**) [32]. Calcium depletion and HCO3^−^ mediated pH neutralization can unfold these aggregates and tightly clumped MUC. The HCO3^−^ ion transportation across the plasma membrane occurred by cystic fibrosis transmembrane conductance regulator (CFTR) channel [33]. Mutation in CFTR channel has been well established with the pathogenesis of cystic fibrosis, which is accompanied with persistently acidic intracellular pH. It leads to 3–4 fold increase in the secretion of MUC, as the packing and aggregation of gel-forming MUC are favored under such acidic condition [33]. Three proteins associated with the secretory MUC include; myristoylated alanine-rich C kinase substrate, calcium-activated chloride channel 3, and cysteine string protein and heat shock protein (HSP) 70. These protein are considered to be highly crucial as their inhibition attenuates secretion of MUC [34]. Synaptotagmins, a family of low affinity Ca^2+^ sensor proteins, are also involved in more than 90% of acute MUC secretion upon extracellular signals [35].

## ALTERED LOCALIZATION OF MUC

### Cytoplasmic localization and its association with disease

**Figure 2:**
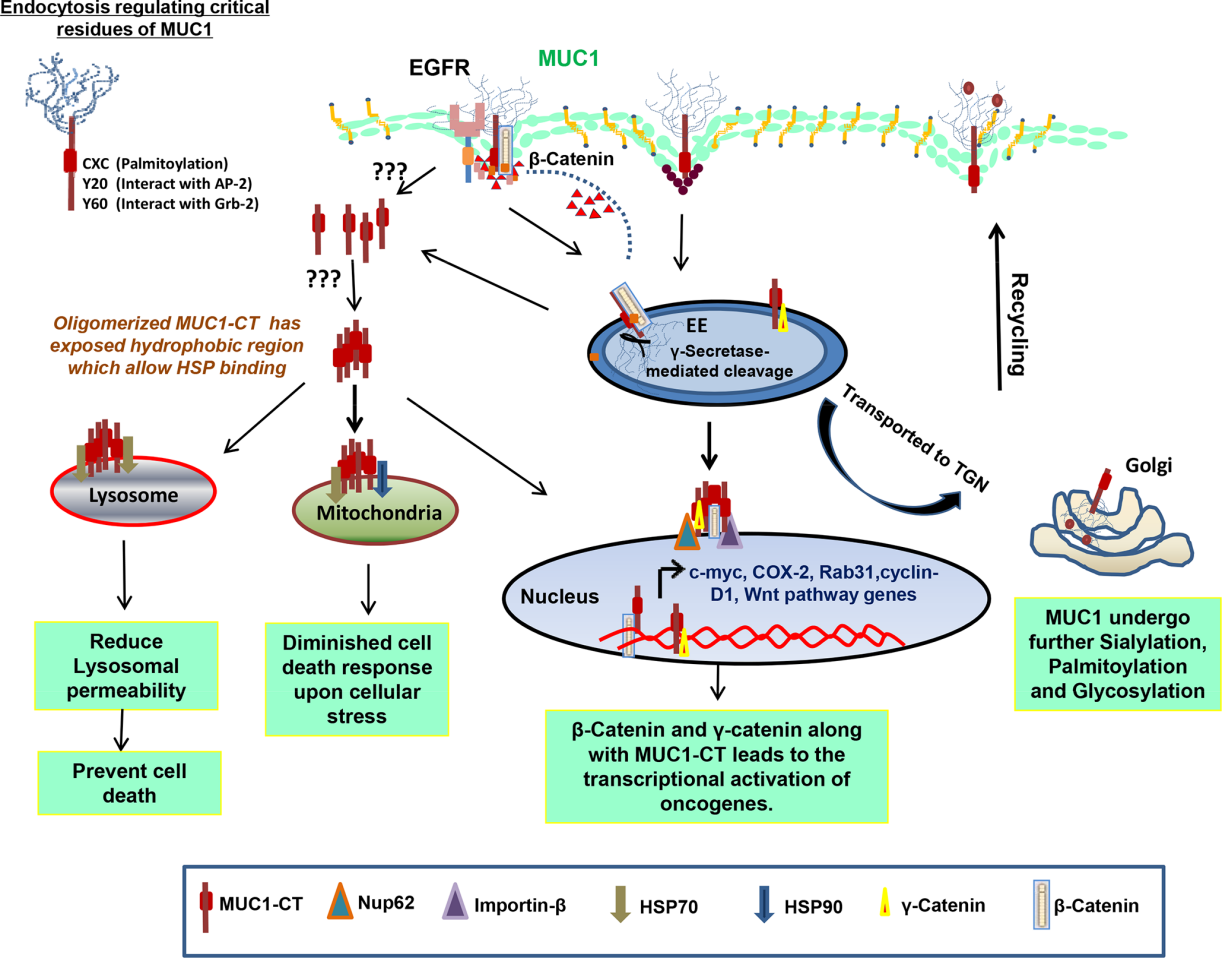
Mechanisms of intracellular transport and sorting of MUC1 MUC1 has demonstrated to be internalization by using clathrin and caveolin-mediated pathway, which is dependent on Rab5a, an early endosome marker. MUC1 has many interacting partners including EGFR family proteins, AP-2, Grb2 and β-catenin. MUC1 possesses a γ-secretase cleavage site and get cleaved in early endosome: **(A)** Cleaved MUC1-C, which is still in contact with β-catenin, travels to nucleus to increase the transcription of various genes that are regulated by the TCF promoter; MUC1-C interacts with heat shock proteins for mitochondrial **(B)** and lysosomal **(C)** translocation resulting in reduced cell death response to DNA damage and cathepsin mediated apoptosis, respectively; **(D)** MUC1, like other glycoproteins, undergoes multiple rounds of sialylation and glycosylation while continuing on the itinerary to the Golgi. MUC1 also has a CQCRRK sequence motif, which undergoes palmitoylation. These post-translational modifications and interacting protein partners play important roles in deciding the fate of MUC1.

In 1992, Ceriani and colleagues conducted immunohistochemistry (IHC) analysis of MUC1 cytoplasmic and membranous expression/localization on 227 breast cancer patients. They found that low cytoplasmic intensity and high cell surface localization of MUC1 correlated with better prognosis of breast cancer patients and survival [36]. This observation was further validated by Rahn *et al.*, who found that increased cell surface MUC1 expression in lower grade and estrogen receptor (ER)-positive tumors have better prognosis, whereas MUC1 cytoplasmic localization in tumors correlated with worse prognosis [37]. Aberrant cytoplasmic MUC1 localization has also been correlated with high-risk papillary thyroid carcinoma [38]. In breast ductal adenocarcinomas, MUC2 and MUC5AC are localized in cytoplasm with granular staining pattern [14, 39, 40], whereas distribution of MUC5B expression changes from apical localization in non-malignant breast cells to cytoplasmic and non-apical localization in malignant ductal breast carcinoma [41]. Similarly, MUC3 cell surface expression has been correlated with poor prognosis, higher grade and negative ER expression in breast carcinoma [42]. These studies clearly demonstrate that, aberrant localization of MUC is associated with cancer pathology [14, 36, 38, 39], and therefore, it is essential to investigate the mechanisms that alter trafficking of MUC among different subcellular compartments. So far, no definite mechanism has been established to understand the elevated intracellular presence of MUC in cancer, but different postulations, specifically for MUC1, have been put forth including; its impaired recycling, altered glycosylation, altered endocytosis and other presumed changes in MUC dynamics (**Fig. 2**), which will be discussed in detail in the next sections.

### Nuclear translocation of MUC1

Reports have shown nucleolar localization of MUC1 in human breast carcinomas but not in normal mammary ductal epithelium (**Fig. 2A**). The cytoplasmic domain of MUC1 (or MUC1-C) is comprised of 58 amino acids of the ECD, 28 amino acids of the TM domain and 72 amino acids of the cytoplasmic tail (CT). MUC1-C does not contain a prototypical monopartite nuclear localization signal (NLS); though it has a positively charged amino acid stretch known as the RRK motif and has been implicated in γ-catenin nuclear localization [43]. A similar potential NLS is also present in MUC16-CT, but it is yet to be investigated for its role in MUC 16-CT nuclear translocation [44]. In breast cancer cell lines, FGF1 has been found to facilitate the targeting of MUC1-C to the nucleus [45]. FGF1 induced the phosphorylation of MUC1 on Tyr46 residue, which increases the interaction between MUC1 and β-catenin, and thus participates in MUC1-C translocation to the cytosol and nucleus [45]. So far, the oligomerization of CQC sequence of MUC1-C has been shown to be obligatory for MUC1-C nuclear translocation. The translocation of the oligomerized MUC1-C into the nucleus is facilitated via its association with importin-β and nucleoporin 62 [46], present on both the cytoplasmic and nucleoplasmic sides of nuclear pore complex, respectively. However, a recent report has indicated that the ectodomain of MUC1 can also translocate to the nucleus, although to different area of the nucleus. MUC1-C localization to the nuclear periphery, nucleolar and in nuclear matrix [47], whereas, the ectodomain of MUC1 or MUC1-N localizes to the nuclear speckles, area which is primarily associated with the complex and crucial process of splicing [48]. Intriguingly, MUC1 post-transcriptionally stabilizes galectin-3 expression in breast cancer cells [49], which was attributed to MUC1 mediated suppression of microRNA-322 expression. Nevertheless, miRNA independent mechanisms could also be involved, as suggested by the presence of MUC1-N in the nuclear speckles. These studies make it evident for us to reconnoiter whether galectin-3 expression is regulated by the MUC1-N mediated splicing process or not.

Altogether, growth factors induced nuclear translocation of MUC1 in conjunction with other oncogenic proteins (such as γ-catenin and β-catenin) provides survival and proliferative advantages to the cancer cells by inducing the transcription of other proteins such as cyclinD1 and c-myc in a TCF/LEF family member-dependent transcriptional upregulation [50].

### Mitochondrial translocation of MUC1

Despite the absence of a classical mitochondrial localization signal, MUC1-C gets localized to the outer membrane of mitochondria by its interaction with cytosolic chaperones such as the HSP70 and the HSP90 [51] (**Fig. 2B**). MUC1-C mitochondrial localization has been correlated with the diminished cell death response to the DNA damage and other cellular stress by inhibiting the release of cell-death causing factors. Cytosolic sequestration of MUC1 exposes its hydrophobic TM domains that facilitate their binding with chaperones, and thus targeting to the mitochondria. Interestingly, Heregulin (HRG), a ligand of EGFR family receptor family, enhances the association between MUC1-C and HSP90 due to autophosphorylation and activation of c-Src in HCT116/MUC1 cells [52] and facilitates the translocation of MUC1-C to the mitochondria. In breast cancer cells, FGF1 plays similar role in the mitochondrial localization of MUC1 using similar molecular mechanism [45]. Taken together, MUC1 translocation to the mitochondria might be negatively regulating the programmed cell death.

### Lysosomal translocation of MUC1

Recent report has also demonstrated lysosome as another subcellular compartment where MUC1-C can localize utilizing similar HSP dependent mechanism as used for mitochondrial localization (**Fig. 2C**) [53]. Loss of HSP70 is associated with increased intracellular Ca^2+^ levels and lysosomal permeability, causing death of pancreatic cancer cells [54]. However, the exact mechanism of such HSP70 mediated protection was not known. In a recent study, Banerjee *et al.* have shown that HSP70 prevents lysosomal permeabilization by its physically interaction with MUC1-C. This association is followed by the lysosomal translocation of MUC1-C [53], which inhibits the release of lysosomal hydrolytic enzymes, particularly, cathepsin B, cathepsin D and cathepsin L. These cathepsins can functions even at a neutral cytosolic pH and have the ability to activate apoptotic effectors such as calpains and caspases to elicit apoptotic processes [55]. However, MUC1 overexpression followed by its cleavage to generate MUC1-C is very well utilized by cancer cells to protect them from harsh apoptotic programs.

Hence, altered localization of MUC1-C to the lysosome followed by its interaction with overexpressed lysosomal HSP70 in pancreatic cancer cells prevents cathepsins mediated cell death response by inhibiting their release from lysosome.

## POST TRANSLATIONAL MODIFICATION AND THE ABERRANT LOCALIZATION OF MUC

### Glycosylation

All mucins contain PTS domains, composed of proline, tyrosine and serine residues and serve as sites for extensive O-linked glycosylation, which contributes up to 80% of their molecular weight and imparts most of their antigenic epitopes [56]. Inhibition of O-glycosylation by 1-benzyl-2-acetamido-2-deoxy-α-d-galactopyranoside (GalNAcα-O-bn) impedes the apical targeting of glycoproteins by inhibiting the docking/fusion of protein carrying vesicles to the plasma membrane, as this inhibitor interferes with the localization of proteins involved in apical trafficking such as, the apical t-SNARE, syntaxin-3 and the raft-associated protein annexin XIIIb [57]. Besides O-glycosylation, MUC also contain potential N-glycosylation sites. For example; MUC13 have seven N-glycosylation sites [58]. N-glycosylation of MUC plays important roles in their folding, sorting, and secretion [59]. Studies have demonstrated that absence of N-glycosylation blocks the apical targeting of glycoproteins, which results in the accumulation of glycoproteins in the Golgi complex of polarized Madin-Darby canine kidney epithelial cells (MDCK) and non-polarized Chinese hamster ovary (CHO) cells and make them proteolytically sensitive [60]. However, due to unresolved issues including that of cell specificity have so far precluded the identification of specific glycan determinants involved in this apical targeting.

It has been observed that epithelial cancers expresses MUC1 with truncated or under-glycosylated glycans, such as the Tn (GalNAcα-) and TF (Galβ1, 3GalNAcα-) antigens [21]. Comparison of the stability of the differentially glycosylated forms of MUC1, derived from normal CHO cells and UDP-glucose-4-epimerase deficient (glycosylation-defective) ldlD cells, revealed that defective glycosylation can significantly re-route MUC1 from the plasma membrane to the degradation pathway. In a parallel experiment, addition of exogenous GalNAc to the culture media resulted in MUC1 stabilization on the cell surface (60% of fully glycosylated MUC1), emphasizing the importance of glycosylation in MUC stability. On the other hand, MUC1 with short glycan structures have shown two-fold higher rate of endocytosis via the hypertonic-media sensitive clathrin-mediated pathway, along with increased intracellular sequestration, as compared to the mature [^35^S] MUC1 [20, 21]. Interestingly, this increased internalization of truncated MUC1 was not followed by its degradation. Apart from clathrin-mediated endocytosis, a separate study has shown that MUC1 can also be endocytosed via macropinocytosis (**Fig. 1**) [22], which suggests the involvement of multiple endocytic pathways in MUC1 internalization. These observations raise questions, such as: whether the alternative pathway of internalization is responsible for increased MUC1 endocytosis and does the mode of internalization for MUC1 change during pathological condition? Answer to these questions can help us to design better strategies against MUC1 targeted antigens.

Further, Razawi *et al* have suggested that membrane-localized and secretory MUC1, both have altered O-glycan core structures, due to the differential pathway of their trafficking [61]. Authors utilized recombinant epitope-tagged MUC1 (MUC1-M), mutants with defective clathrin-mediated endocytosis (MUC1-M-Y20,60N) and mutants with recycling defects (palmitoylation-defective MUC1-M-CQC/AQA). Intriguingly, CQC/AQA mutants showed significantly reduced level of transits to the TGN and accumulation in endosomal compartments. Analysis of shed MUC1 ectodomain subunit of the CAC/AQA mutant revealed change in the core glycan structures from sialylated core 1 (MUC1-M, wild-type) to core 2 glycans on the non-recycling CQC/AQA mutant. Interestingly, the O-glycoprofile of the non-recycling CQC/AQA mutant exhibits similarity to the core 2 glycoprofile on a secretory MUC1 which enters only once to the Golgi complex [61]. On the other hand, O-glycoprofile of the MUC1-M-Y20, 60N mutant resembles the wild-type phenotype with dominant core 1 expression.

### Sialylation

Similar to altered glycosylation, sialylation patterns of MUC also differed in cancer cells as compared to the normal healthy cells. Previous reports have demonstrated the presence of immature form of episialin (MUC1) on the cell surface under cancerous conditions. The immature episialin is convertible to mature form by addition of sialic residues through consecutive internalization and routing of the MUC1 to the TGN (**Fig. 2D**) [62]. The half-life of MUC1 at the plasma membrane has been calculated to be 16–24 h [63], and each round of sialylation takes around 2.5 hours [62], which suggests that MUC1 possibly undergo recycling about 9-10 times. The multiple rounds through TGN generate abnormally high levels of sialylation [62], and have been correlated with increased metastatic potential of cancer cell [64–67]. Similarly, elevated sialyl-Lewisx (SLex) epitope has been associated with the poor prognosis of colon cancer patients [68]. In addition, due to the presence of SLex epitopes on selectin ligands, the unusual higher levels of sialylation of MUC could play a critical role in the invasion and metastasis of cancer cells [69] and leukocyte extravasation during inflammation.

### Palmitoylation

MUC1 has the CQCRRK sequence motif, which can be palmitoylated. This motif in MUC1 is present at the boundary of TM domain and cytoplasmic tail, and its palmitoylation has been correlated with MUC1 plasma membrane retention. This MUC1 retention is achieved by regulating MUC1 recycling at the apical surface without interfering or altering the rate of its endocytosis [17]. Studies have also revealed the importance of tyrosine residues at position 20 and 60 in the MUC1-CT domain, in the process of endocytosis [70]. The Y20N mutation in association with the CQCRRK motif inhibit the interaction of MUC1 with the adaptor proteins AP-1 and AP-2, whereas the Y60N mutation inhibits MUC1 association with another adaptor protein called Grb2. Transfection of CHO cells with double mutant; AQA mutant and Y20N mutant caused MUC1 accumulation in Rab11-positive recycling endosomes, apparently due to reduced affinity of the mutant for AP-1, and thus poor recycling. On the other hand, transfection of Y20N mutant showed reduction in the rate of endocytosis and internalization, however, the subcellular distribution of MUC1 remain unchanged. Altogether, these findings suggest that palmitoylation plays an important role in MUC1 routing from endosomes to the plasma membrane. Palmitoylation of MUC1 cytoplasmic tail might be inducing conformational changes which could interfere with the interactions between MUC1-CT and endocytosis regulating proteins. It is noteworthy that palmitoylation is an important modification for the trafficking of a number of receptors, ion channels and signaling proteins [71]. The precise mechanism by which palmitoylation is regulating membrane trafficking is not as clear. The palmitoylation and depalmitoylation status permit H-Ras and G protein subunits to transduce signals from intracellular compartments like Golgi complex [72]. From a clinical perspective, it may be worthwhile to design targeted therapies against palmitoylation regulating enzymes.

## DIFFERENTIAL INTERACTION OF MUCINS WITH PROTEINS UNDER PATHOLOGICAL CONDITION

Additional factors may also modulate the localization of MUC under cancerous conditions. One example is the differential MUC interactions due to galectin-3 overexpression (**Fig. 1**) [73]. Galectin-3 is predominantly expressed as a cytosolic protein in epithelial tissues, though it can also localize to the nucleus, mitochondria and extracellular regions [74]. In pancreatic cancer, silencing of galectin-3 has shown to enhance the cell surface interaction between MUC1 and EGFR in the absence of EGF stimulation and reduced the rate of endocytosis of MUC1-EGFR complex which leads to the noticeable cell surface localization of MUC1. However, presence of EGF stimulation leads to the nuclear translocation of EGFR without affecting MUC1 cell surface and MUC1-CT nuclear localization [75]. Upon rescuing this knockdown effect by recombinant galectin-3, discernible redistribution of MUC1 from the cell surface to the cytoplasm was observed [75]. Therefore, galectin-3 overexpression in cancer could possibly be related to the frequently observed intracellular retention of MUC1.

## MODULATION OF SUB-CELLULAR PROTEIN TRAFFICKING BY MUCINS

Due to the loss of polarity in cancer, MUC (MUC1 and MUC4) localize all over the cell surface, instead of restricted confinement at the apical surface. This allows them to interact with cell surface proteins such as the EGFR family members, which normally exist at the basolateral sides of polarized cells [76, 77]. MUC4 has shown to interact with HER2/ErbB2 in ovarian and pancreatic cancers [76, 78]. MUC4-ErbB2 complex lead to the activation of various signaling pathways leading to cell proliferation and survival through stimulation of p38 MAPK phosphorylation [79]. In the absence of the soluble ligand, the MUC4-ErbB2 complex leads to ErbB2 phosphorylation, which in turn, leads to the phosphorylation of the ErbB2-ErbB3 heterodimer in the presence of neuregulin [80]. MUC4 did not demonstrate interaction with ErbB3 in polarized cells, but loss of polarity facilitates MUC4-ErbB3 interaction.

The tradeoff between phosphorylation and glycosylation (O-GlcNAc) is known to regulate intracellular trafficking of EGFR [81]. MUC1 is known to interact with EGFR at plasma membrane of non-polarized breast epithelia which resulted in increased EGFR internalization, reduced lysosomal degradation and increased EGFR recycling back to the plasma membrane [77]. Likewise, MUC4 has also shown to interact with the other EGFR family member, HER2, via its EGF-like motifs located at the juxtamembrane domains [82]. The EGF-like motif is also present in other MUC like MUC17 which has been implicated in the pathogenesis of colonic inflammation and cancer, and can presumably initiates EGFR mediated oncogenic signaling. Interestingly, activated EGFR phosphorylates YEKV motif in MUC1-CT to induce MUC1 interaction with c-Src and β-catenin. MUC1-CT also has a γ-secretase cleavage motif and the cleavage by γ-secretase results in the release of intracellular MUC1-CT to regulate MUC1 mediated cellular proliferation [83]. The MUC1-CT and E-cadherin both compete for β-catenin binding due to the loss of cellular polarity [45, 84].

Like EGFR family members, β-catenin also resides at the lateral side of the cell. The loss of polarity allows β-catenin to interact with the SAGNGGSSL motif present in MUC1-C and the loss of E-cadherin-mediated cell-to-cell interaction at MUC1 positive sites. Under normal conditions, β-catenin interacts with the similar S*XXXXX*SSL motif of E-cadherin, which is required for the maintenance of the adherent junction. This interaction between β-catenin and MUC1 is regulated by EGFR mediated phosphorylation of the crucial tyrosine residues present on MUC1-CT [47]. Additionally, phosphorylation of the serine residue in SPYEKV sequence by glycogen synthase kinase 3β (GSK3β), a site adjacent to the β-catenin binding motif inhibits the interaction between MUC1 and β-catenin [85]; whereas c-Src mediated phosphorylation of the tyrosine residue in that same SPYEKV site enhances their interaction [86]. MUC1 shows binding affinity to the Armadillo repeats and the non-repetitive COOH-terminal region of β-catenin [87]. MUC1 and β-catenin, once in complex, mitigates GSK-3β phosphorylation of β-catenin and translocate to the nucleus to transcriptionally activate various genes implicated in increased carcinogenic potential and metastasis [88]. In breast and colon cancers, HRG stimulation facilitates the binding between MUC1-CT and γ-catenin, allowing MUC1 to function as a vehicle for γ-catenin nuclear translocation [43]. These findings indicate that MUC1-CD has crucial functions in integrating signals from the EGFR and Wnt signaling pathways. Unlike in MUC1 and MUC4, the RTK binding motif is not present in MUC16-CT [89]. However, MUC16 secretion is influenced by EGF stimulation through phosphorylation of MUC16-CT [90]. MUC16 knockdown in ovarian cancer cell lines caused increased cytoplasmic localization of β-catenin and E-cadherin, and was linked with greater cellular motility and invasiveness [89]. In agreement, reduction of MUC16 expression has been related with advanced ovarian cancer [91]. Taken together, these studies pointed towards the possibility that the interactions between MUC16, E-cadherin and/or β-catenin permit MUC16 to modulate various signaling pathways.

Bitler *et al.* found evidence that MUC1 has regulatory functions in the trafficking and nuclear activity of EGFR [47]. Presence of MUC1 showed enhance interaction between EGFR and phosphorylated RNA polymerase II, which implies that MUC1 can impact the association of EGFR with transcriptional machinery at the promoter region, as the loss of MUC1 reduces the occupancy of EGFR at the cyclin D1 promoter region [47]. Besides controlling such inter-molecular interactions, MUC1-C also regulates Rab31 expression, which is an early endosome protein belonging to the subfamily of small GTPase Rab5 [92]. MUC1-C and estrogen receptor form a complex at the Rab31 promoter and are responsible for the transcriptional activation of Rab31. According to this study, patients who express MUC1-C and Rab31 are resistant to tamoxifen treatment indicating the possible involvement of these two molecules in determining the efficacy of tamoxifen therapy [92].

## THERAPEUTIC PERSPECTIVES

The altered MUC localization and interactome under pathological conditions provide new avenue for the therapeutic intervention. Multiple studies have investigated the potential of two well characterized MUCs, MUC1 and MUC4, as therapeutic targets by restraining their subcellular localization.

### MUC1-C inhibitors

Recently, protein-trafficking pathways have been exploited to enhance anti-cancer drug sensitivity of melanoma cells. Melanoma cells showed 10-fold increase in the sensitivity to cis-diaminedichloroplatinum II (cDDP, cis-platin), carboplatin and other anti-cancer drugs upon depletion of the vacuolar protein sorting 33A or the cappuccino protein [93], which strongly signpost the idea that therapeutic targeting of protein trafficking molecules can increase the drug sensitivity of cancer cells.

GO-201 and GO-202 are two small peptides that recognize the CQC motif in the amino terminal of MUC1-C responsible for the translocation of MUC1-C to various sub-cellular organelles [94]. Both GO-201 and GO-202 showed anti-tumorigenic functions *in vitro* and in xenograft models [94]. Targeting the endocytic vesicles to compartment specific localizations can also be accomplished by modulating the expression of critical mediators such as Rab proteins. Rab5 has been shown to participates in MUC1 internalization [22], and as mentioned previously, Rab31 overexpression induces MUC1-C expression [92]. Rab expression profiles are modulated under different disease conditions, such as Rab25 expression is altered in breast and colorectal carcinomas [95, 96], and induce the invasive ability of cancer cells by interfering with the endosomal trafficking of cell adhesion proteins such as β-intergrins [97, 98]. Small molecular inhibitors that target promoters of these Rab and other trafficking proteins could be designed to reroute MUC to the degradation pathways. Other small molecular inhibitors like geldanamycin and 17-(allylamino)-17-demethoxygeldanamycin, significantly diminished FGF1-induced MUC1 interaction with HSP90, which as a consequence, obstructed MUC1 targeting to the mitochondria [45].

### MUC4 effects on HER2 internalization

MUC4 stabilizes HER2 on the plasma membrane by inhibiting its internalization in pancreatic cancer [76], whereas MUC1 induces the internalization of EGFR and directs it to the nucleus for transcriptional upregulation of oncogenic factors [47]. Presence of MUC4 and MUC1-C has also been related with Herceptin resistance in breast cancer [99, 100]. One of the mechanism by which Herceptin functions is by binding to the extracellular domain of HER2, and inducing its internalization for lysosomal mediated degradation [101]. Therefore, by inhibiting MUC and HER2 interaction by designing peptides against their interacting motifs could increase the efficacy of Herceptin therapy. Recent study has shown that administration of GO-203 downregulates the levels of phospho-p27 and cyclin-E, which abrogates Herceptin resistance in breast cancer [102]. As MUC4 is one of the most differentially expressed proteins in pancreatic cancer and has been associated with the Herceptin resistance in breast cancer; the failure of HER2 trial in pancreatic cancer could possibly be attributed to MUC4 aberrant overexpression, which requires further investigation.

## SUMMARY AND CONCLUSIONS

MUCs are the major macromolecular components of epithelial mucus and have been incriminated in the pathogenesis of various diseases. Their mislocalization has been well associated with the pathobiology of several cancers such as, breast and colorectal cancer. Under normal condition, MUC are localized predominantly on the apical surface, but loss of polarity allows them to extend all over the cell surface and modulate their interactome. Several unique domains present in MUC play crucial role in determining these interactions. Mislocalization of MUC also facilitates MUC interactions with other novel proteins like catenin and translocate to different subcellular compartments. Though many conjectures, including altered glycosylation, sialylation, and differential protein-protein interactions, have been made to answer altered localization of MUC, the exact mechanism has not been explored and need immediate attention for therapeutic interventions.

## FUTURE DIRECTIONS

Post-translational MUC modifications needed for membrane targeting can also be modulated by regulating the expression and kinetics of critical enzymes, so that MUC can be directed to the degradation pathways, rather than to the cell surface. This strategy has been clinically utilized to generate a number of peptide-based inhibitors [94]. A recent report indicated that a covalent linkage between a glycosylated MUC1-derived glycopeptide and a Toll-like receptor agonist could elicit strong humoral and cellular immune responses [103]. Technological advancements, particularly in mass spectrometry, have enabled the characterization of the entire structure of O-glycans. It would be interesting to determine the structural differences between O-glycans and N-glycans in MUC expressed under normal and pathological conditions and to correlate them with changes in their intracellular trafficking and altered localization. It will not only give us deeper insight into MUC biology, but will also help us design novel therapeutic strategies to treat cancers. In addition, structural differences arise as a result of altered glycans could be responsible for the altered mucin interaction with other proteins such as Her-2 and β-catenin which further strengthen the rationale to investigate MUC trafficking and its altered subcellular localization.

Besides these alteration, other standing questions need to be answered like does MUC1 enter the nucleus following the classical pathway dependent on importins and nucleoporins; does it go through a non-classical pathway similar to β-catenin? How is the palmitoylation of CQC motif of MUC1-C regulated? Under what stimuli, the fate of the subcellular targeting of MUC1-C is determined? What functions MUC1-N have in the nucleus? Is there any correlation between altered splicing and MUC1 overexpression in cancer? How is MUC1-N translocation to the nucleus regulated and/or mediated? The outcome of CQC palmitoylation or its nuclear/mitochondrial/lysosomal translocation may be very different depending on various unknown parameters and need to be explored. Nuclear localization of MUC16 has also been speculated because of the presence of potential NLS signal in MUC16-CT, which needs to be validated due to its well-established oncogenic role in multiple cancers.

## Abbreviations

TGN: Trans-Golgi network
ECD: Extracellular domain
TM: Transmembrane
CT: Cytoplasmic tail
NLS: Nuclear localization signal
HSP: Heat shock protein
HRG: Heregulin
Nup62: Nucleoporin 62
EGFR: Epidermal growth factor receptor
FGF1: Fibroblast growth factor
Y: Tyrosine residue
AP-2: Adaptor protein-2
